# Emergence of bimodal cell population responses from the interplay between analog single-cell signaling and protein expression noise

**DOI:** 10.1186/1752-0509-6-109

**Published:** 2012-08-24

**Authors:** Marc R Birtwistle, Jens Rauch, Anatoly Kiyatkin, Edita Aksamitiene, Maciej Dobrzyński, Jan B Hoek, Walter Kolch, Babatunde A Ogunnaike, Boris N Kholodenko

**Affiliations:** 1 Dept. of Pharmacology and Systems Therapeutics, Mount Sinai School of Medicine, New York, NY, 10029, USA; 2 Systems Biology Ireland, University College Dublin, Belfield, Dublin 4, Ireland; 3 Dept. of Anatomy, Pathology, and Cell Biology, Thomas Jefferson University, Philadelphia, PA, 19107, USA; 4 Dept. of Chemical and Biomolecular Engineering, University of Delaware, Newark, DE, 19716, USA

## Abstract

**Background:**

Cell-to-cell variability in protein expression can be large, and its propagation through signaling networks affects biological outcomes. Here, we apply deterministic and probabilistic models and biochemical measurements to study how network topologies and cell-to-cell protein abundance variations interact to shape signaling responses.

**Results:**

We observe bimodal distributions of extracellular signal-regulated kinase (ERK) responses to epidermal growth factor (EGF) stimulation, which are generally thought to indicate bistable or ultrasensitive signaling behavior in single cells. Surprisingly, we find that a simple MAPK/ERK-cascade model with negative feedback that displays graded, analog ERK responses at a single cell level can explain the experimentally observed bimodality at the cell population level. Model analysis suggests that a conversion of graded input–output responses in single cells to digital responses at the population level is caused by a broad distribution of ERK pathway activation thresholds brought about by cell-to-cell variability in protein expression.

**Conclusions:**

Our results show that bimodal signaling response distributions do not necessarily imply digital (ultrasensitive or bistable) single cell signaling, and the interplay between protein expression noise and network topologies can bring about digital population responses from analog single cell dose responses. Thus, cells can retain the benefits of robustness arising from negative feedback, while simultaneously generating population-level on/off responses that are thought to be critical for regulating cell fate decisions.

## Background

Development, growth and homeostasis of multi-cellular organisms depend on the ability of individual cells to convert noisy, analog signals into clear, yes-or-no cell fate decisions, such as apoptosis, proliferation and differentiation. One way that cells make such decisions is through the use of signal transduction systems that sense the strength of an analog input signal, and then convert it into one of several distinct activity states, such as “on” or “off” output states of highly ultrasensitive or bistable systems [1-3]. For example, various mitogen concentrations can cause bistable activation of cyclin-dependent kinases to drive cell cycle transition decisions [4-6]. Theoretical studies have shown that signaling networks containing positive or double negative feedback loops [3], opposing modification enzymes exhibiting saturation kinetics [1] and multi-site modification cycles [2,7] can exhibit digital (bistable or ultrasensitive) behavior. However, not all networks that contain such motifs will necessarily exhibit digital behavior; such behavior arises from the cell’s precise tuning of quantitative, spatiotemporal aspects of the network. Indeed, the signal transduction network connecting epidermal growth factor (EGF) to activation of extracellular signal-regulated kinase 1/2 (ERK) contains many elements that potentially can lead to switch-like behavior. However, previous single cell studies in different mammalian cell lines have reported both graded [8,9] and “all-or-nothing” [10] EGF-induced ERK activation responses. One determinant of whether signaling is graded or switch-like is the spatial localization of signal processing proteins [11].

Under idealized conditions of cell-to-cell homogeneity, experimental techniques such as immunoblotting that measure average population responses may be able to detect all-or-none signaling responses, as long as the cell-to-cell variability in response activation thresholds are negligible [12]. However, it is becoming clear that the fundamental processes of transcription and translation are inherently stochastic, and give rise to significant cell-to-cell variability in protein levels [13-20]. The primary stochastic factors are (i) the rate of transcription, which is burst-like due to the low number (two) of genes for a particular protein in a cell [21,22] and (ii) the number of proteins produced per mRNA, which is random due to competition between ribosomes and RNase for the mRNA [13,23,24]. Protein degradation also contributes to expression noise, but usually to a lesser extent, since protein copy numbers are typically large enough to dampen the comparatively small stochastic fluctuations in degradation rate. Thus, even genetically identical cells show substantial variations in protein and mRNA abundance, and as a result, may also show differences in their signaling responses [25]. Because of such heterogeneity in protein abundance, population average measurements are not sufficient for investigating “all-or-nothing” responses; single-cell measurement techniques capable of capturing the dynamics of digital signal transduction are needed [12].

Here, we use flow cytometry to measure EGF-induced, single-cell ERK activation responses in a HEK293 cell population. We observe bimodal response distributions in cell populations that are usually thought to indicate switch-like behavior in single cells. Surprisingly, an ERK cascade signaling model incorporating negative feedback and a graded, analog single cell dose response is shown to be consistent with the observed population responses. Our model analysis suggests that such a conversion of analog responses in single cells to digital responses at the population level is due to protein abundance variability, which gives rise to a broad distribution of ERK pathway activation thresholds and RasGTP levels. Thus, bimodal response distributions do not necessarily imply digital single cell signaling; such distributions can arise from the interplay between protein expression noise and negative feedback-mediated, analog single-cell responses.

## Results

### Analyses of ERK responses to EGF in individual cells and populations

**Figure 1:**
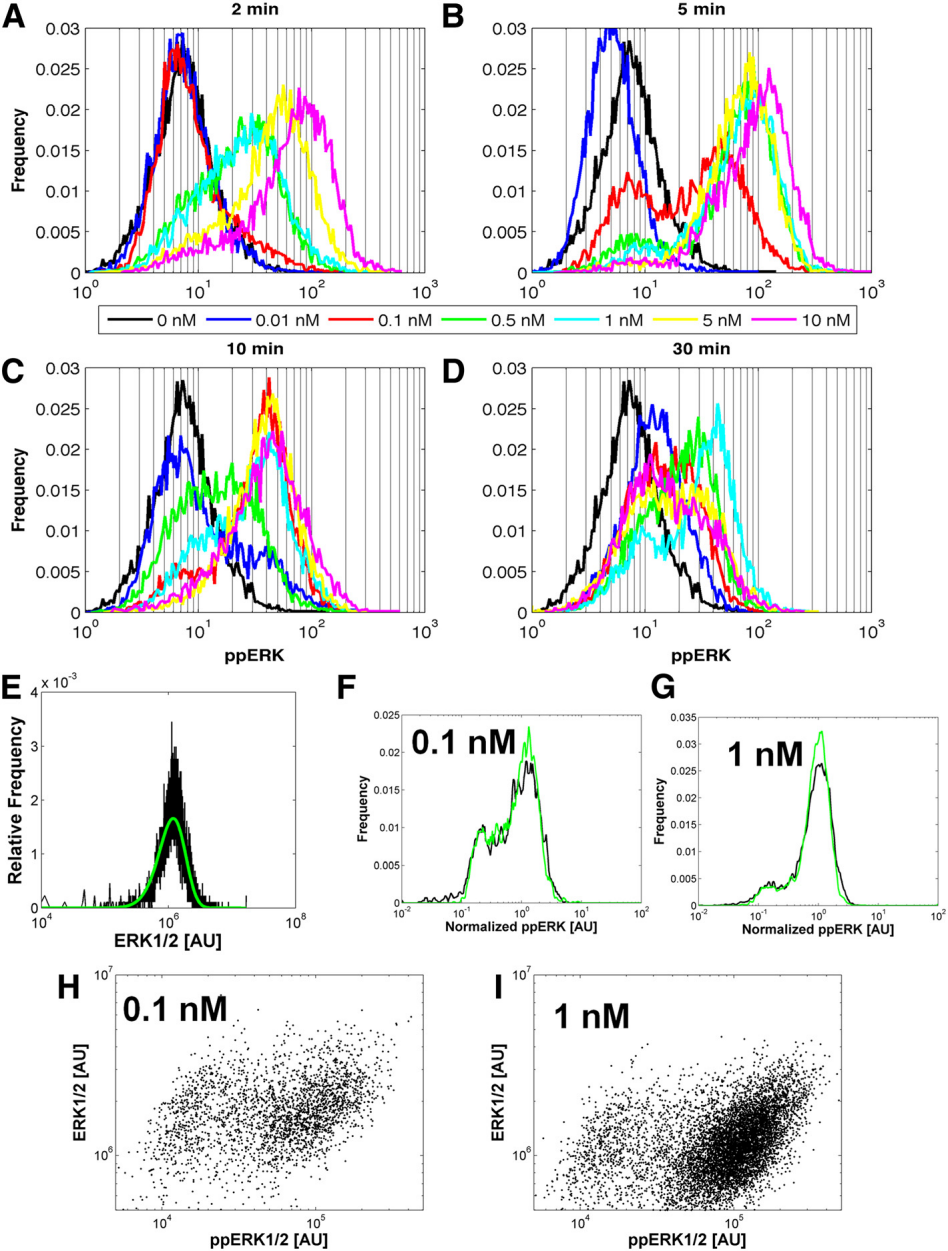
**Cell population dose and dynamic response of ppERK to EGF. A-D**. Each panel corresponds to a fixed time after EGF stimulations: 2 min (**A**), 5 min (**B**), 10 min (**C**) and 30 min (**D**). In each panel, the different colors correspond to different EGF doses as indicated by the visual legend. Each distribution is compiled from 10,000 individual HEK293 cell responses as measured by flow cytometry, and is representative of between three and six independent experiments. Events were gated based on forward and side scatter to exclude debris, dead cells, and cell clusters. The x-axis is the magnitude of activated ERK (ppERK) in arbitrary fluorescence units, and the y-axis is the frequency of observing a particular level of fluorescence in a cell. **E.** Total ERK abundance data. The best-fit gamma distribution curve is depicted by the green line, while the black line shows experimental data. Data are representative of five independent experiments, and were fit to a gamma distribution. Mean and standard deviation of the fit parameters are *k* = 5.4; θ = 2.7 × 10^5^ [AU]. **F-I**. Cells were stimulated with either 0.1 nM (**F,H**) or 1 nM (**G,I**) EGF for five minutes, and then analyzed by flow cytometry to measure ppERK and total ERK levels simultaneously. In **F-G**, black curves correspond to ppERK distributions, and green curves correspond to normalized distributions where ppERK levels in each cell were divided by the total ERK signal intensity in the same cell. To compare the green and black curves on the same axis, intensities for each distribution are divided by their respective mean. In the **H-I** dot plots, ppERK levels are on the x-axis, whereas total ERK levels are on the y-axis.

We used a flow cytometry-based phosphorylation assay (FCPA) [26] to determine the kinetics and dose response of ERK activation by EGF in HEK293 cells. We show that population averages obtained from FCPA results correspond well to traditional Western blot measurements of activated (dually phosphorylated) ppERK levels in cell populations (Additional file 1: Figure S1). However, the FCPA also reveals how individual cells contribute to this collective population response (Figure 1A-D; Additional file 1: Figure S2). The increase in mean values of ppERK was dose-dependent after two minutes of EGF stimulation, suggesting that analog signaling has occurred in individual cells. However, a fraction of cells contain ppERK levels similar to those of the basal state. We refer to this feature of the distribution as a shoulder. Although the height of this shoulder decreases with increasing EGF dose, its position remains unchanged, indicating a dose-dependent fraction of cells failing to activate ERK. At five minutes after EGF stimulation, the ppERK distribution is unambiguously bimodal, implying digital “on-off” behavior. Higher EGF doses increase the fraction of cells with high ppERK (”ERK-on”) at the expense of the “ERK-off” population. Thus, in a dose-dependent manner, EGF increases the probability that a cell will have ERK turned on. At later time points, a bimodal distribution persists at some EGF doses, while data from other doses show “shouldering” patterns similar to the behavior at 2 minutes. Thus, the EGF-induced ERK response on the population level is complex consisting of both analog and digital elements.

Next, we investigated how cell-to-cell variability in total ERK abundance affects the ppERK responses. Measurements of the total ERK distribution by flow cytometry, as expected, revealed substantial cell-to-cell variability in total ERK levels (Figure 1E). The data are well-approximated by a gamma distribution, which has been postulated by others to be a good representation of cell-to-cell variability in protein levels (Figure 1E-green line) [22,27-31]. We then stimulated cells with 0.1 and 1 nM EGF for 5 minutes and measured both ppERK and ERK levels simultaneously (Figures 1F-I). Normalizing the ppERK levels by the amount of total ERK in each individual cell does not change the variance of “ERK-off” population (Figures 1F-G—compare green to black lines). This is most likely because measurement variability is dominant at these low ppERK levels, and normalizing by total ERK levels does not correct for measurement variability. Normalizing the ppERK levels by total ERK levels does reduce the variability of the “ERK-on” population, but does not change the fraction of cells in the “ERK-on” and “ERK-off” populations (Figures 1F-G). This assertion is reinforced by the fact that ppERK levels in both the “ERK-off” and “ERK-on” populations span the entire spectrum of total ERK levels (Figures 1H-I). Moreover, there is significant positive correlation between total ERK and ppERK levels in both the ERK-off and ERK-on populations (Figures 1H-I). Thus, although cell-to-cell variability in ERK abundance contributes to ppERK response variability, it does not control bimodality, raising the question of what other factors contribute to the observed bimodality.

### Stochastic, dynamic modeling explanation of the data

**Figure 2:**
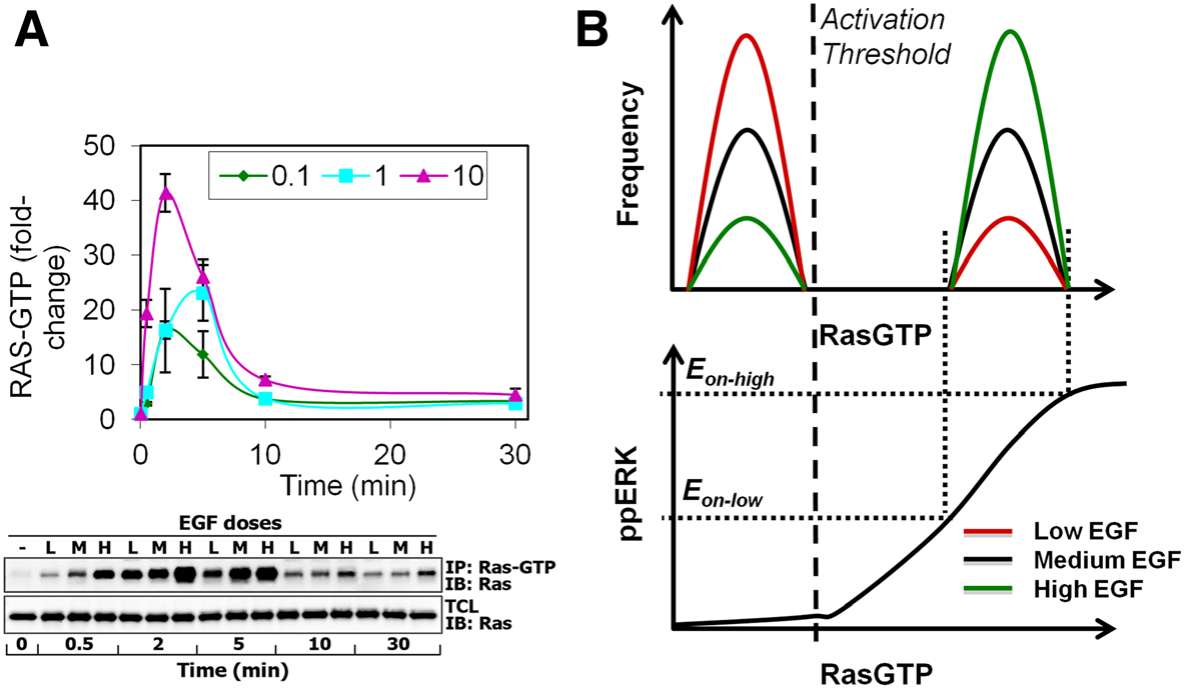
**RasGTP Dynamics. A.** HEK293 cells were stimulated with 0.1 nM (low, L), 1 nM (medium, M) or 10 nM (high, H) EGF for the indicated times and then cell lysates were assayed for RasGTP as described in “Materials and Methods” section. IP denotes the pull-down fraction, TCL denotes total cell lysate, and IB denotes immunoblot. Each data point corresponds to the average of three independent experiments, and error bars correspond to 90% confidence intervals. Data were normalized by dividing by basal (no EGF) RasGTP levels. **B.** A bimodal RasGTP distribution as would be obtained from a bistable RasGTP model for low, medium and high levels of EGF, and how it would map onto a dose–response relationship between RasGTP and ppERK.

EGF activates the small GTPase Ras, which activates ERK downstream of the Raf and MEK kinases. Although we were not able to measure GTP-bound active Ras (RasGTP) by flow cytometry, the population average dose and dynamic responses were assayed via pull-down and Western blotting, and then quantified (Figure 2A). These population average data show a rapid rise and dose-dependent peak in RasGTP levels after EGF stimulation, followed by a fast decline. Although the most direct interpretation of these RasGTP responses (where the population mean changes as a function of time and EGF dose, Figure 2A) is a unimodal RasGTP distribution, a recent study suggested that in T lymphocytes, a positive feedback between RasGTP and its activator guanine exchange factor Son of Sevenless (SOS) leads to bistability and hysteresis in Ras activation [32-34]. If Ras activation was also bistable in HEK293 cells, then two distinct RasGTP populations would exist with high mean and low mean RasGTP levels (Figure 2B). Stimulation by EGF would only affect the relative fraction of cells in the two populations, but not their means. Since under basal conditions ppERK levels are negligible (Additional file 1: Figure S1A), the low mean RasGTP population would not contribute to ERK activation, implying that there is a threshold above which RasGTP levels cause ERK activation (Figure 2B). If we assume a simple sigmoidal dose–response relationship between RasGTP and ppERK levels (typical in MAPK cascades—reviewed in [35]), then a defined high mean RasGTP population would induce a defined high mean ppERK population with boundaries *E*_*on-low*_ and *E*_*on-high*_ (Figure 2B). However, the flow cytometry data in Figure 1A-D show that when clear bimodality is present, *E*_*on-low*_ and *E*_*on-high*_ are different for various high mean ppERK populations. Thus in HEK293 cells, our single cell ppERK signaling data seem to be inconsistent with a bistable RasGTP model.

**Figure 3:**
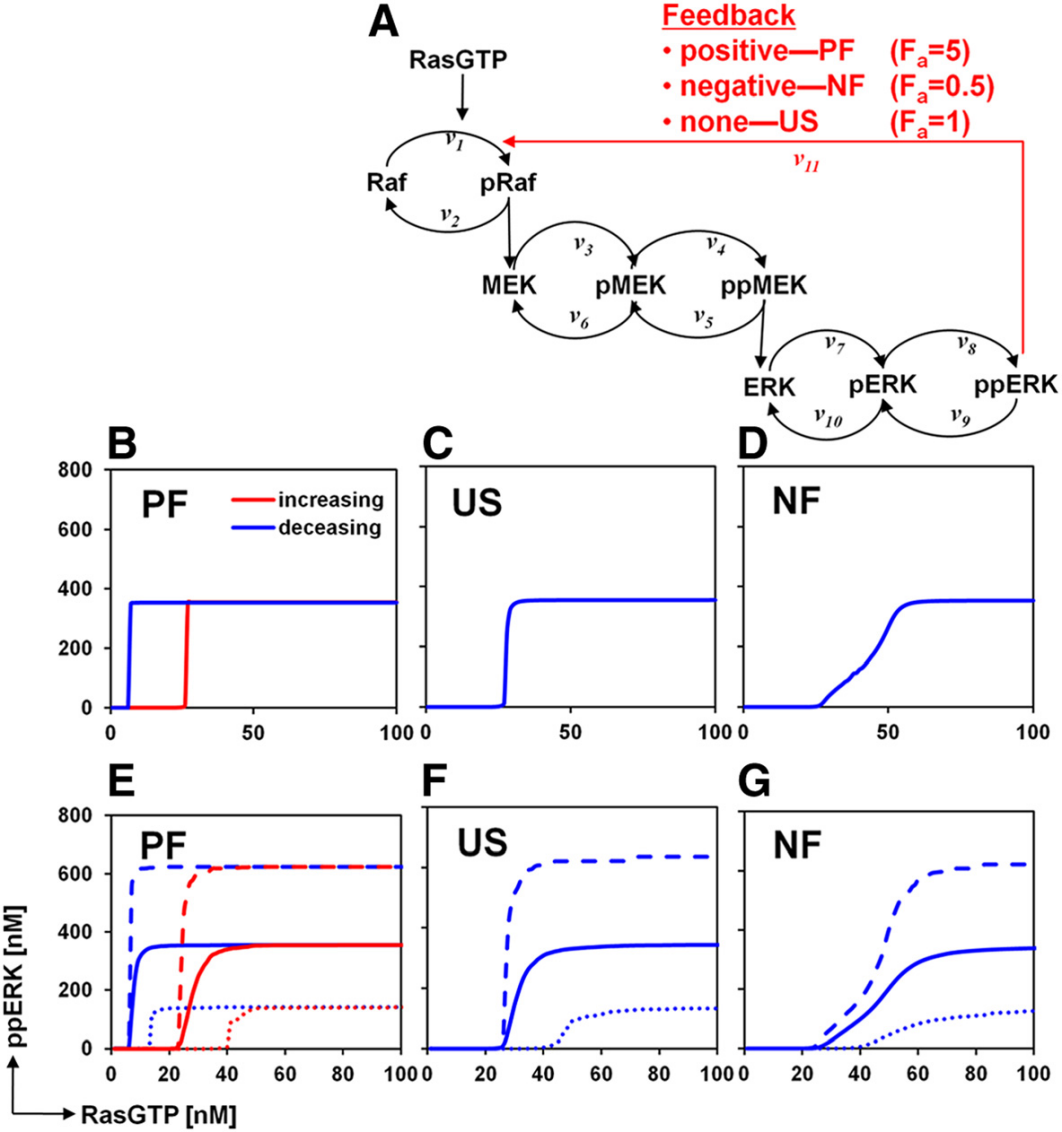
**Modeling and analysis of single cell characteristics of the ppERK dynamics and dose response. A.** Schematic of a mechanistic model of ERK activation and its steady-state response properties. The positive feedback (*PF*), no-feedback, ultrasensitive (*US*), and negative feedback (*NF*) models have the feedback strength *F*_*a*_ set to different values (5, 1 and 0.5, respectively). The input is RasGTP, and the output is ppERK. **B-D.** Steady-state, deterministic input/output response curves for the PF, US and NF models. **E-G.** Steady-state, cell population input/output response curves for the PF, US and NF models. In **B-G**, red denotes increasing input from low levels, while blue denotes decreasing input from high levels. When only one color is shown, there is no difference between the increasing and decreasing input curves. Dashed lines indicate the 95^th^ percentile of all simulations, and dotted lines indicate the 5^th^ percentile.

If the RasGTP response to EGF is unimodal, then how might these mixed analog-digital responses emerge from salient features of the MAPK/ERK cascade? At the single cell level, dynamic responses are encoded by the pathway topology and reaction kinetics. Therefore, we examined different configurations of the MAPK/ERK cascade for their ability to reproduce the experimentally observed behavior. Specifically, we sought topologies where simulations showed that (i) distributions of active ERK display bimodal/shouldering behavior with increasing EGF dose, and (ii) the “ERK-on” population mean increases with increasing EGF dose at early time points, but decreases with time at constant EGF dose. To explore this, we used a previously developed mechanistic model that relates active Ras to ppERK [36], and investigated *in silico* the ability of different network topologies to reproduce our experimental observations (Figure 3A). By changing the feedback strength parameter (*F*_*a*_) in this model, we created three different topologies: positive feedback (PF; *F*_*a*_ = 5), ultrasensitive (US; *F*_*a*_ = 1), and negative feedback (NF; *F*_*a*_ = 0.5), all of which have been experimentally observed for MAPK cascades under various circumstances (reviewed in [35]).

#### Steady-state analysis

First, we characterized the steady-state input–output behavior of these three models by changing the input (RasGTP) from zero to 100 nM at 1 nM increments and allowing the system reach a steady-state between each step change. Then, we reversed the stimulation, this time changing the input from 100 to zero nM. The PF model exhibits bistability/hysteresis, whereas the US and NF models do not (Figure 3B-D). In fact, due to the inherent properties of a negative feedback loop coupled with a kinase amplifier module, the NF model exhibits a smooth, analog input–output relationship [37-40]. However, the NF model also exhibits a threshold of ERK activation at low RasGTP levels as a result of the multi-tier, multi-site phosphorylation structure of the MAPK/ERK cascade [2].

These deterministic simulations correspond to input–output curves for an average cell. To incorporate stochastic fluctuations in reaction rates, we applied the Gillespie algorithm to integrate the differential equation. However, these solutions did not appreciably change the steady-state dose responses (data not shown), indicating that under these conditions and model parameters, reaction rate fluctuations do not constitute a significant source of signaling variability. This is most likely due to the relatively high abundance of the MAPK/ERK cascade components.

We therefore explicitly included protein expression variability in the models. We first investigated whether the gamma distribution provides a generally valid model for the distribution of protein levels, as others have suggested [22,27-31]. We found that there is good agreement between gamma distribution fits and both experimental and stochastic simulation data from the literature (Additional file 1: Figure S3A-E) [22]. Next, we performed our own stochastic simulations using a simple protein expression model where a gene can be active or inactive, an active gene can produce mRNA, mRNA can produce protein, and both mRNA and protein can degrade, all with first order kinetics. We then analyzed the resulting distribution of steady-state protein abundance obtained from multiple independent simulations under 6400 different parameter conditions (see Additional file 1: Figure S3 legend). For most conditions, the steady-state protein abundance distribution is well represented by a gamma distribution (Additional file 1: Figure S3F-G). Therefore, for the steady-state analysis we sampled total levels of Raf, MEK and ERK from a gamma distribution, and computed the dose response curves for 1000 cells, each cell having different, sampled levels of Raf, MEK and ERK (Figures 3E-G). The means of these stochastic, steady-state response curves (solid lines) have the same qualitative features as the deterministic curves, and the PF model remains bistable. However, there is substantial cell-to-cell variability in the dose responses. The RasGTP levels eliciting half-maximal ppERK responses vary significantly, as do the maximum ppERK levels. According to these results, stochastic variability in protein expression is a major contributor to steady-state, cell-to-cell signaling variability, inducing a wide distribution of ERK activation thresholds.

#### Analysis of transient responses

**Figure 4:**
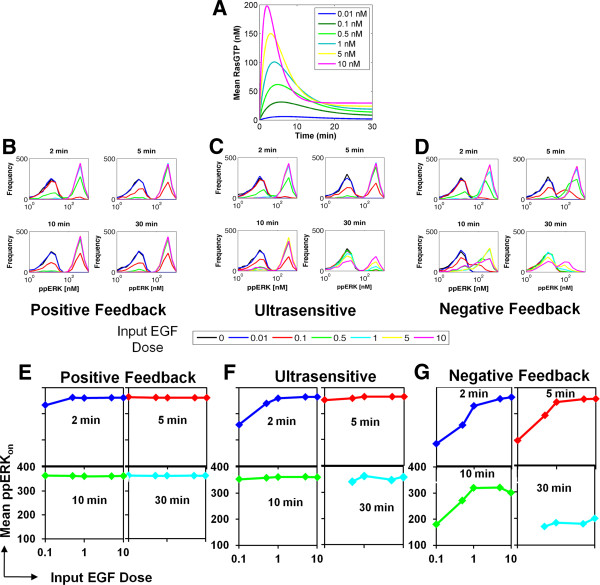
**Simulations of ppERK dynamics in cell populations. A.** Simulated RasGTP dynamics for different EGF doses. Simulations were done as described in the “Materials and Methods” section. **B-D.** Simulated dose and dynamic ppERK responses for the PF (**B**), US (**C**) and NF (**D**) models. To facilitate comparison of these simulations with the experimental data, normally distributed noise with mean and standard deviation of 10 nM was added to the raw simulation data. (**E-G**) Dynamics and dose response of the ERK-on population mean for the PF (E), US (F), and NF (G) models. Simulated population responses were parsed into ERK-on and ERK-off populations based on a cutoff of 100 nM.

To simulate the dynamic behavior of ppERK, we first specified the RasGTP input kinetics, according to the unimodal RasGTP distribution hypothesis discussed above. Experimental data show that in EGF-stimulated HEK293 cells, RasGTP levels peak between 1–5 minutes after EGF stimulation and then, approximately 10 minutes later, decay to a steady-state value that is slightly higher than basal RasGTP levels (Figure 2A and [41]). Moreover, increasing the EGF dose increases the peak magnitude of RasGTP levels, and shortens the rise time. We incorporated these experimentally observed trends into a simple mathematical model (see Methods), and obtained simulated RasGTP dynamics. We then used these simulated dynamics as input to the MAPK/ERK cascade model for determining the ppERK dynamic and dose responses (Figure 4A). To incorporate cell-to-cell variability in Ras levels, we sampled the *peak* RasGTP values from a gamma distribution whose mean increases with increasing input magnitude (with fixed shape parameter *k*—see Methods).

Using these RasGTP dynamics, we then investigated which models (NF, PF or US) reproduce the experimental observations described above. As expected, the PF and US models show bimodal population behavior because of their switch-like input–output responses (Figure 4B-C). But surprisingly, so too does the NF model, despite exhibiting an analog input–output relationship (Figure 4D). This bimodality in the NF model is due to the wide range of ERK activation thresholds introduced by protein expression variability (Figure 3G), combined with variability in EGF-induced RasGTP levels. Thus, all three topologies exhibit time and dose-dependent bimodality or “shouldering”. However, only the NF model simulations, and not those of the US or PF models, reproduce proper behavior of the ERK-on population mean, namely that the mean increases as a function of dose at short times (Figure 4E-G; Figure 1), and decreases as a function of time at a particular EGF dose (Figure 4E-G; Additional file 1: Figure S2).

We conclude that for the realistic parameter values used here, the NF model with protein expression variability is most consistent with experimental data. To examine if this conclusion holds over a wide range of parameter values, we employed parameter sensitivity analysis (see Methods and Additional file 1: Figure S4). This analysis showed that models with negative feedback preferentially demonstrated the experimentally observed signaling characteristics over the examined parameter ranges (Additional file 1: Figure S4). Yet, we cannot rule out the possibility that positive feedback and ultrasensitive systems may also exhibit the experimentally observed behavior. Indeed, sensitivity analysis also showed that under some rare parameter conditions, the mean ppERK levels in the ERK-on population increase as a function of dose at short times for the PF and US models (Additional file 1: Figure S4A,C). One mechanism that may lead to this PF and US model behavior is if the ppERK activation kinetics were slow, such that the behavior at 2 and 5 min post EGF stimulation were due to transient effects, rather than a pseudo-steady state phenomenon. Yet, for PF models, simulated ppERK signaling remains high over the 30-minute time course (Additional file 1: Figure S4B,D), rather than returning closer to basal levels as the experimental data show (Additional file 1: Figure S2). Thus, the ERK cascade model with negative feedback seems to be the most consistent with our experimental observations over a wide range of parameter values.

### Test of the negative feedback prediction

**Figure 5:**
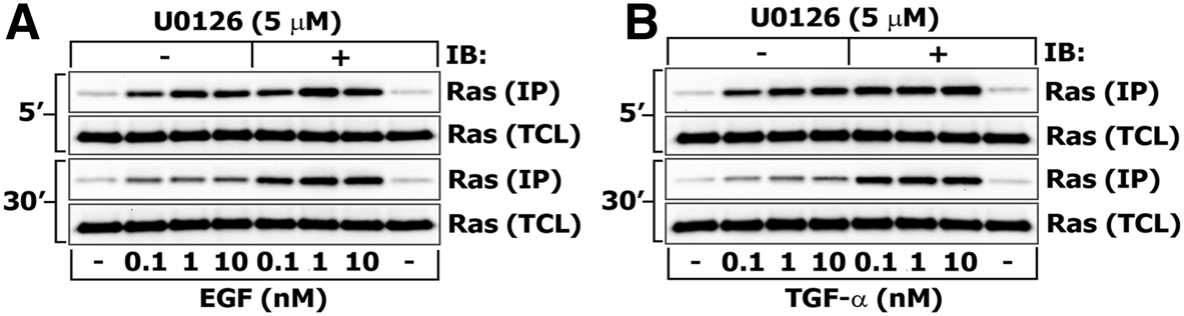
**Confirming the presence of negative feedback.** HEK293 cells were pretreated with 5 μM U0126 or vehicle alone (DMSO) for 30 min prior to stimulation with 0.1 nM, 1 nM or 10 nM of EGF (**A**) or TGFα (**B**) for 5 or 30 minutes. Control cells were left unstimulated (−). Total cell lysates were assayed for activated RasGTP as described in “Materials and Methods” section. IP denotes the pull-down fraction, TCL denotes total cell lysate, and IB denotes immunoblot.

Although the preceding analysis suggests that in our HEK293 cell system the most likely net feedback strength from ERK is negative, parameter sensitivity analysis showed that ultrasensitive or positive feedback systems might also account for such data, albeit in rare circumstances. If the feedback were negative, blocking ERK activity should increase the activation of upstream elements, such as RasGTP. Therefore, we measured the dynamic and dose response of RasGTP with and without the MEK inhibitor U0126, and found that blocking ERK activation increased RasGTP levels, confirming the presence of strong negative feedback (Figure 5A). Although positive feedback and ultrasensitivity have been observed in various MAPK cascades (reviewed in [35]), in HEK293 cells the major feedback regulation is negative, confirming the predictions of the modeling. Notably, this feedback is less significant at five minutes after EGF stimulation, when the RasGTP response is saturated and ppERK levels are at their peak, implying that either this feedback is slow (which may introduce instability and oscillations under certain conditions [42]), or perhaps that there are alternative negative feedback mechanisms.

To investigate whether alternative negative feedback mechanisms may explain the weak feedback effects at 5 minutes post-stimulation, we repeated the U0126 experiment with the EGF receptor ligand TGFα. Although both EGF and TGFα activate the EGF receptor and induce receptor endocytosis, EGF preferentially targets the receptor to multi-vesicular bodies and lysosomal degradation, while TGFα enhances receptor recycling and surface availability [43,44]. Thus, it is possible that EGF-induced receptor degradation or sequestration may be influencing our results. We found that the TGFα-induced RasGTP levels do not differ from those induced by EGF in the presence or absence of the MEK inhibitor U0126 over a 30-minute time course (Figure 5B). Therefore we conclude that negative feedback from ERK seems to dominate trafficking-mediated effects.

## Discussion

**Figure 6:**
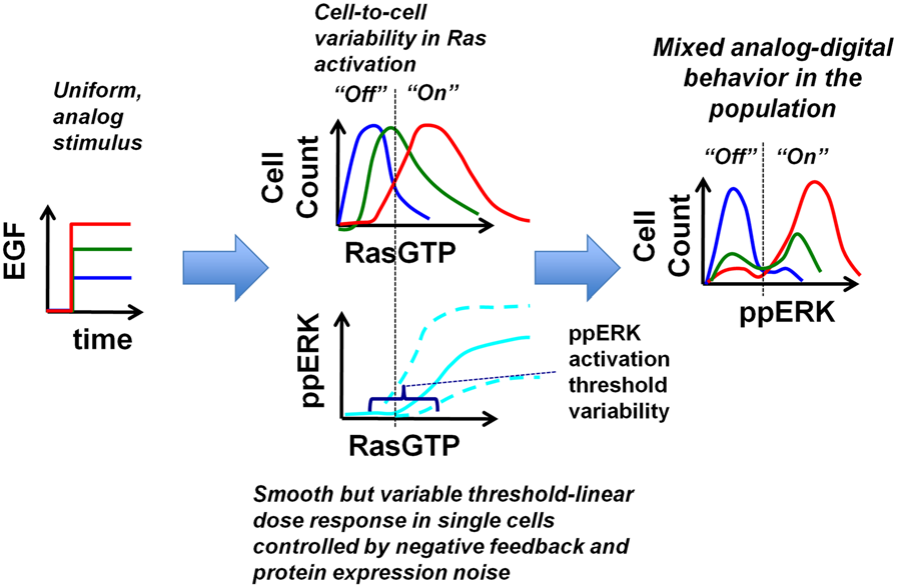
**Conversion of Analog Inputs Into Bimodal Responses by a Negative Feedback System Combined with Protein Expression Noise.** An analog EGF stimulus (blue, green, and red correspond respectively to small, medium, and large stimulation magnitudes) induces variable but dose-dependent RasGTP responses in the cell population due to expression variability in the EGF pathway proteins. RasGTP responses are converted into ppERK responses in single cells according to a threshold-linear response governed by negative feedback (NF model). However, variability in RasGTP levels coupled with variability in ERK activation thresholds creates bimodal active ERK distributions at the population level despite the analog input and linear dose response at the single cell level.

We have studied EGF-induced signal transduction to ERK in single HEK293 cells, finding that the conversion of an analog signal at the single cell level to an apparent digital response at the population level can be mediated by a combination of cell-to-cell variability in protein expression and a pathway design that incorporates negative feedback (Figure 6). A uniform step increase in EGF concentration causes a wide distribution of RasGTP levels due to cell-to-cell heterogeneity in protein expression. Cell-to-cell heterogeneity in protein expression also causes significant variability in the sigmoidal dose response relationship between RasGTP and ppERK, and in particular, in the ppERK activation threshold (Figure 3G and Figure 6). Because cell-to-cell variability in RasGTP levels can span the range of ERK pathway activation thresholds, the pathway is activated to various degrees in individual cells. A distribution of ppERK levels ensues across the cell population. The mean of the ppERK distribution depends on EGF dosage and agrees with results obtained from Western blots. Despite the fact that the negative feedback smooths the RasGTP/ppERK dose–response relationship, a threshold for ppERK activation persists. This threshold element further enhances cell-to-cell variability in ppERK levels, and results in bimodal responses at the population level. Thus, the resulting bimodal distribution relies on a combination of a threshold behavior and a linear ppERK increase followed by saturation behavior with increasing EGF dose (Figure 3G). Surprisingly and counterintuitively, bimodality does not require switch-like behavior at the single-cell level, but can arise from cell-to-cell variability in protein expression and a pathway activation threshold. Thus, cells can retain the robustness benefits offered by negative feedback [37-40], while generating on/off responses at the cell population level that are thought to be critical for cell fate decisions.

Our observations are unlikely to be caused by a fraction of cells simply not binding ligand. First, under our experimental conditions (~10^6^ cells/mL), at the lowest ligand dose (0.01 nM), the ratio of EGF molecules to cells is approximately 1000, making it very unlikely that a cell does not encounter a ligand molecule. Second, for nearly all EGF doses, a significant fraction of cells is in the “ERK-on” population at some point in time, indicating that most cells have been activated and therefore had bound ligand.

How might cells still generate reliable signals despite protein expression noise? One possibility is that cells have a reliable fold-change response of ppERK from basal levels, and that downstream of ppERK cells employ systems that sense fold-changes rather than absolute levels. In fact this fold-change scenario has recently been shown to be the case. In cells stably expressing ERK2-YFP from the endogenous promoter, EGF stimulation led to widely varying maximum nuclear ERK2-YFP accumulation, with a coefficient of variation (CV) of approximately 0.3 [15]. However, normalizing the maximum nuclear ERK2-YFP signal by the basal levels of ERK2-YFP in the same cell, which yields fold-change responses, lowers the CV by approximately 3-fold [15]. This is consistent with our observed effects of total ERK abundance variability on the total variance of ppERK in the ERK-on population (Figure 1F-G). To sense these fold-changes, rather than absolute levels, a cell may use a type-1 incoherent feedforward loop (I1-FFL), where an input X activates both an intermediate Y and the output Z, but Y represses Z [45]. Such a network structure may in principle be downstream of ppERK (X), which causes the immediate-early expression of multiple genes including *c-fos*, which can mediate general transcriptional repression perhaps even of itself [46,47].

Although protein expression noise is certainly a hindrance to some biological functions, and evolution has selected for mechanisms such as the I1-FFL that allow a cell to deal with this noise, there are potential benefits of and perhaps even essential functions for such noise. Tissue homeostasis may in fact require protein expression variability. Consider that there is no protein expression variability, and all cells that are involved with, for instance, hematopoiesis, respond identically to the various proliferation and differentiation cues. The body needs to produce, from the hematopoietic stem cells, a balance between the lymphoid and myeloid progenitors. If all the hematopoietic stem cells responded identically, then it would be nearly impossible for the body to maintain a finely tuned balance between the production of these two lineages. The same logic applies to the further differentiation of lymphoid and myeloid progenitors into various other downstream cell types, such as megakaryocytes, erythrocytes, B cells, T cells, and natural killer cells, where finely tuned control of differential cell-fate decisions is even more critical. Thus, it is likely that without protein expression noise-induced phenotypic variability, homeostasis of hematopoiesis, and probably other tissues, would not be possible. This logic argues for a conceptual model whereby growth factor concentration, in tissues, controls the probability a cell will choose a particular fate.

## Conclusions

It is commonly thought that the existence of bimodal signaling behavior on the population level is indicative of so-called digital behavior (such as all-or-none switches) of the underlying signaling network in single cells. Our work demonstrates that this is not necessarily the case; protein expression noise coupled with nonlinear network dynamics can bring about digital population responses from analog single cell dose responses. In particular, we show that a network combining an activation threshold and strong negative feedback also robustly displays such bimodal population behavior due to cell-to-cell variability in protein expression levels. This system retains the benefits of robustness arising from negative feedback, while simultaneously generating population-level on/off responses thought to be critical for cell fate decisions. Overall, the results extend our understanding of the amazing behavioral complexity that can be displayed by even small molecular networks [48].

## Methods

### Cell culture

Human Embryonic Kidney 293 (HEK293) cells were obtained from the American Type Culture Collection (Manassas, VA). Cells were maintained in a humidified 5% CO_2_ incubator at 37°C and cultured in Dulbecco's modified Eagle's medium/F-12 supplemented with 10% fetal bovine serum (Life Technologies-Invitrogen, Carlsbad, CA) and penicillin-streptomycin solution (100 μg/ml, Thermo Fisher Scientific).

### Flow cytometry

HEK293 cells were serum starved for 16 hours before the experiment. The cells were then lifted (by scraping or trypsinization), washed twice with serum-free medium (containing soybean trypsin inhibitor in the case of tryptic lifting), allowed to equilibrate for 30 minutes, and stimulated with EGF (Sigma-Aldrich, St. Louis, MO). We verified that the bimodal ppERK behavior was not affected by cell detachment (Additional file 1: Figure S5). After EGF stimulation for the desired time interval, cells were fixed with 2% paraformaldehyde (Sigma-Aldrich) for 10 minutes at 37°C, and then cooled on ice. After centrifugation, the cells were permeabilized in ice-cold 90% methanol (Sigma-Aldrich) for 30 minutes. The cells were then washed by centrifugation and 5x10^5^ cells were resuspended in 90 μL incubation/blocking buffer (0.5% BSA in PBS) for 10 minutes. The cells were then incubated for 60 minutes in the dark at room temperature with phospho-ERK1/2 (T202/Y204) mouse mAb (E10) Alexa 488 Conjugate for active ERK and ERK1/2 rabbit mAb (4695) detected by secondary staining with an anti-rabbit Alexa 647-conjugate (Cell Signaling Technologies, Beverley, MA). The cells were washed by centrifugation with PBS and resuspended in 0.5 mL of PBS. The samples were then analyzed with a Becton-Dickinson FACSCalibur or on an Accuri C6. For each sample, 10,000 events (cells) were analyzed. Data were processed using FlowJo^™^ software (Tree Star, Inc.) and MATLAB^™^ (The Mathworks). Post-gating by forward and side scatter was performed to remove events corresponding to dead cells, debris, and cell clusters (i.e. doublets). As controls we stained cells with non-specific, isotype-matched control antibodies (also obtained from Cell Signaling). We verified the specificity of the antibodies (Additional file 1: Figure S1).

### Western blotting

The above procedure for cell preparation was followed, but instead of fixing cells in paraformaldhyde, cells were lysed and processed for Western blotting analysis as described previously [49,50]. RasGTP pull-downs were performed as described previously [49,50].

### Mechanistic model simulations

MATLAB and the function *ode15s* was used to simulate a previously developed, ordinary differential equation-based ERK cascade model [36], which is described in detail in Tables 1 and 2. The function *gamrnd* was used to generate realizations of peak RasGTP, Raf, MEK, and ERK levels for individual “cells” in the stochastic simulations according to the gamma distribution

(1) $$f\left(N\right)=\frac{N^{\left(k-1\right)}e^{-\frac{N}{\theta }}}{\theta^{k}\Gamma \left(k\right)}$$

where *N* specifies a protein level, *k* is the shape parameter, and θ is the scale parameter. We specified the *k* (shape) parameter of each gamma distribution as 5.4, as was measured for total ERK (see Figure 1E), assuming roughly similar expression regulation. Since the mean of a gamma distribution is equal to *k*θ, the θ parameter of each gamma distribution was changed as needed to attain the desired distribution mean (see Table 1 for values of mean protein levels).

**Table 1:** Kinetic description of the ERK signaling cascade

**N**	**Reaction**	**Rate**	**Kinetic**
			constant*
1	MAP3K → pMAP3K	$v_{1}=\frac{k_{1}^{\mathrm{cat}}\cdot \left[Ras-GTP\right]\cdot \left[MAP3K\right]/K_{m1}}{\left(1+\left[MAP3K\right]/K_{m1}\right)}\cdot g\left(F_{a}\right)$	$k_{1}^{\mathrm{cat}}=0.2$; $K_{m1}=50$
2	pMAP3K → MAP3K	$v_{2}=\frac{V_{\max2}\cdot \left[pMAP3K\right]/K_{m2}}{\left(1+\left[pMAP3K\right]/K_{m2}\right)}$	$V_{\max2}=5$; $K_{m2}=50$
3	MAP2K → pMAP2K	$v_{3}=\frac{k_{3}^{\mathrm{cat}}\cdot \left[pMAP3K\right]\cdot \left[MAP2K\right]/K_{m3}}{\left(1+\left[MAP2K\right]/K_{m3}+\left[pMAP2K\right]/K_{m4}\right)}$	$K_{3}^{\mathrm{cat}}=1$; $K_{m3}=130$
4	pMAP2K → ppMAP2K	$v_{4}=\frac{k_{4}^{\mathrm{cat}}\cdot \left[pMAP3K\right]\cdot \left[pMAP2K\right]/K_{m4}}{\left(1+\left[MAP2K\right]/K_{m3}+\left[pMAP2K\right]/K_{m4}\right)}$	$k_{4}^{\mathrm{cat}}=5$; $K_{m4}=50$
5	ppMAP2K → pMAP2K	$v_{5}=\frac{V_{\max5}\cdot \left[ppMAP2K\right]/K_{m5}}{\left(1+\left[ppMAP2K\right]/K_{m5}+\left[pMAP2K\right]/K_{m6}+\left[MAP2K\right]/K_{i1}\right)}$	$V_{\max5}=250$; $K_{m5}=100$
6	pMAP2K → MAP2K	$v_{6}=\frac{V_{\max6}\cdot \left[pMAP2K\right]/K_{m6}}{\left(1+\left[ppMAP2K\right]/K_{m5}+\left[pMAP2K\right]/K_{m6}+\left[MAP2K\right]/K_{i1}\right)}$	$V_{\max6}=250$; $K_{m6}=100$; $K_{i1}=80$
7	MAPK → pMAPK	$v_{7}=\frac{k_{7}^{\mathrm{cat}}\cdot \left[ppMAP2K\right]\cdot \left[MAPK\right]/K_{m7}}{\left(1+\left[MAPK\right]/K_{m7}+\left[pMAPK\right]/K_{m8}\right)}$	$k_{7}^{\mathrm{cat}}=1$; $K_{m7}=50$
8	pMAPK → ppMAPK	$v_{8}=\frac{k_{8}^{\mathrm{cat}}\cdot \left[ppMAP2K\right]\cdot \left[pMAPK\right]/K_{m8}}{\left(1+\left[MAPK\right]/K_{m7}+\left[pMAPK\right]/K_{m8}\right)}$	$k_{8}^{\mathrm{cat}}=20$; $K_{m8}=50$
9	ppMAPK → pMAPK	$v_{9}=\frac{V_{\max9}\cdot \left[ppMAPK\right]/K_{m9}}{\left(1+\left[ppMAPK\right]/K_{m9}+\left[pMAPK\right]/K_{m10}+\left[MAPK\right]/K_{i2}\right)}$	$V_{\max9}=380$; $K_{m9}=10$
10	pMAPK → MAPK	$v_{10}=\frac{V_{\max10}\cdot \left[pMAPK\right]/K_{m10}}{\left(1+\left[ppMAPK\right]/K_{m9}+\left[pMAPK\right]/K_{m10}+\left[MAPK\right]/K_{i2}\right)}$	$V_{\max10}=50$; $K_{m10}=18\;K_{i2}=100$
11	*Feedback*	$g\left(F_{a}\right)=\frac{\left(1+F_{a}\cdot {\left(\left[ppMAPK\right]/K_{a}\right)}^{2}\right)}{\left(1+{\left(\left[ppMAPK\right]/K_{a}\right)}^{2}\right)}$	$K_{a}=100$; $F_{a}=5;1;0.5\left(\text{PF};\text{US};\text{NF}\right)$

* Maximal rates, Michaelis and catalytic constants are expressed in [nM/s], [nM], and [s^-1^], respectively. Total protein concentrations are [MAPK3]_total_ = 200nM, [MAPK2]_total_ = 200nM, and [MAPK]_total_ = 360nM.

**Table 2:** Ordinary differential equations for the ERK signaling cascade model

	
$\frac{d\left[MAP3K\right]}{dt}$	$v_{2}-v_{1}$
$\frac{d\left[pMAP3K\right]}{dt}$	$v_{1}-v_{2}$
$\frac{d\left[MAP2K\right]}{dt}$	$v_{6}-v_{3}$
$\frac{d\left[pMAP2K\right]}{dt}$	$v_{3}+v_{5}-(v_{4}+v_{6})$
$\frac{d\left[ppMAP2K\right]}{dt}$	$v_{4}-v_{5}$
$\frac{d\left[MAPK\right]}{dt}$	$v_{10}-v_{7}$
$\frac{d\left[pMAPK\right]}{dt}$	$v_{7}+v_{9}-(v_{8}+v_{10})$
$\frac{d\left[ppMAPK\right]}{dt}$	$v_{8}-v_{9}$
$\frac{d\left[RasGTP\right]}{dt}$	$\frac{K_{1}}{\tau_{1}}\text{exp}\left(-\frac{t}{\tau_{1}}\right)+\frac{K_{2}}{\tau_{2}}\text{exp}\left(-\frac{t}{\tau_{2}}\right);RasGTP\left(t\right)=K_{1}\left(1-e^{-t/\tau_{1}}\right)+K_{2}\left(1-e^{-t/\tau_{2}}\right)$

K’s and τ’s are determined as described in Methods.

To estimate the parameters for the RasGTP dynamics, which are described by a simple exponential rise and decay model (see Table 2 for differential equations), we used least squares optimization to ensure that desired initial magnitude (*I*_*o*_), peak magnitude (*I*_*max*_), time-to-peak (τ_*max*_), time-to-inflection (τ_*infl*_), time-to-steady-state (τ_*ss*_), and steady-state magnitude (*I*_*ss*_) of the RasGTP dynamics matches well to that which the model prescribes. Additional file 1: Figure S6 describes these RasGTP dynamics metrics graphically. As there are four unknown parameters in the RasGTP dynamics model (Table 2-K_1_, K_2_, τ_1_, τ_2_), we need four equations, which we take as the following (their origin is described immediately below):

(2) $$0=\left(I_{o}+\left(K_{1}+K_{2}\right)-I_{\mathrm{ss}}\right)w_{1}$$

(3) $$0=\left(\frac{K_{1}}{\tau_{1}}\exp\left(-\frac{\tau_{\max}}{\tau_{1}}\right)+\frac{K_{2}}{\tau_{2}}\exp\left(-\frac{\tau_{\max}}{\tau_{2}}\right)\right)w_{2}$$

(4) $$\begin{array}{ll} 0= & \left(I_{o}+K_{1}\left(1-\exp\left(-\frac{\tau_{\mathrm{ss}}}{\tau_{1}}\right)\right)\right)\\ & \left(+,K_{2},\left(1-\exp\left(-\frac{\tau_{\mathrm{ss}}}{\tau_{2}}\right)\right),-,1.01,\cdot ,I_{\mathrm{ss}}\right)w_{3} \end{array}$$

(5) $$\begin{array}{ll} 0= & \left(I_{o}+K_{1}\left(1-\exp\left(-\frac{\tau_{\max}}{\tau_{1}}\right)\right)\right)\\ & \left(+,K_{2},\left(1-\exp\left(-\frac{\tau_{\max}}{\tau_{2}}\right)\right),-,I_{\max}\right)w_{4} \end{array}$$

where *w*_*i*_ corresponds to a weight for optimization purposes (all *w*’s are 1 except for *w*_*2*_ which is 100). Eq. 2 specifies the proper steady-state magnitude; Eq. 3 specifies that the 1^st^ derivative at the time-to-peak is zero; Eq. 4 specifies the proper magnitude at the time-to-steady state (defined as 1% of the true steady-state value—see Additional file 1: Figure S6); and Eq. 5 specifies the proper peak magnitude. The following constraints are placed on this optimization problem:

(6) $$0>-\frac{K_{1}}{{\tau_{1}}^{2}}\exp\left(-\frac{\tau_{\max}}{\tau_{1}}\right)-\frac{K_{2}}{{\tau_{2}}^{2}}\exp\left(-\frac{\tau_{\max}}{\tau_{2}}\right)$$

(7) $$0>\frac{K_{1}}{\tau_{1}}\exp\left(-\frac{\tau_{\mathrm{infl}}}{\tau_{1}}\right)+\frac{K_{2}}{\tau_{2}}\exp\left(-\frac{\tau_{\mathrm{infl}}}{\tau_{2}}\right)$$

Eq.6 specifies that there is a maximum at the time-to-peak (2^nd^ derivative less than zero) and Eq. 7 specifies that the 1^st^ derivative is negative at the inflection point (RasGTP is decreasing towards the steady-state value). Mean peak RasGTP levels (*I*_*max*_) were increased to simulate increasing input, and were linearly spaced between 10 nM and 200 nM using 6 points (10, 48, 86, 124, 162, and 200 nM), which correspond to EGF doses (in nM) of 0.01, 0.1, 0.5, 1, 5, and 10. Following the trends of the experimental data in Additional file 1: Figure 2A and [41], peak times for RasGTP (τ_*max*_) were sampled linearly between 7 min and 2 min (7, 6, 5, 4, 3, 2), with 7 min corresponding to the lowest peak RasGTP level (EGF dose). Also, we took τ_*ss*_ as 10 min, *I*_*ss*_ as 15% of *I*_*max*_ realizations, *I*_*o*_ as 0, and τ_*infl*_ as (τ_*max*_ + τ_*ss*_)/2.

All code is available upon request.

### Parameter sensitivity analysis

Five hundred different parameter sets were generated via latin hypercube sampling (MATLAB function *lhsdesign*) from a 23-dimensional uniform distribution that spans +/− 1 order of magnitude around each nominal parameter value (taken from Table 1 with the exception of *F*_*a*_—the feedback strength). For each of these parameter sets stochastic simulations were performed as described above. Briefly, total protein and RasGTP levels were sampled from a gamma distribution and 500 individual cell responses were simulated for each parameter set and feedback condition (negative, ultrasensitive with no feedback, positive). The results of these simulations were then analyzed for three features: the “analogicity” of the ERK-on population, the “transience” of the ERK-on population, and bimodality. The analogicity of a particular feedback/parameter set combination was calculated as follows, and is illustrated in Additional file 1: Figure S4A. First, the ERK-on population was defined by those cells having ppERK levels over 200 nM. Then, the mean ppERK levels in the ERK-on populations were calculated for those that contained greater than 10 cells. The analogicity of a given time point is defined as the maximum ERK-on population mean minus the minimum (as compared across EGF doses). The analogicity of a feedback/parameter set combination is the sum of the 2 and 5 minute time point analogicities. The 10 and 30 minute time points are left out because these show very little analogicity in the experimental data (Figure 1 and Additional file 1: Figure S2). Parameter sets showing zero analogicity were discarded as inconsistent with experimental data. The transience of a particular feedback/parameter set combination is defined for a particular EGF dose as follows, and is pictorially illustrated in Additional file 1: Figure S4B. First, the ERK-on population was defined as described above for analogicity, and any EGF dose where the ERK-on population did not exist for all time points was not used for further transience calculations. The transience of an individual EGF dose is the mean of the ERK-on population at 2 and 5 minutes minus that at 10 and 30 min. The transience of a feedback/parameter set combination is the sum over those from the individual EGF doses. Bimodality was evaluated via Hartigan’s Dip Test [51,52]. MATLAB code for this test was downloaded from http://www.nicprice.net/diptest/. The result is a p-value associated with the hypothesis test that the empirical distribution of interest is unimodal as opposed to the alternative that it is not. We rejected the null hypothesis at the 0.05 level of significance. The bimodal fraction for a particular feedback/parameter set combination is defined as the number of non-unimodal distributions divided by the total number of dose/time point combinations. Parameter sets showing no bimodality were discarded as inconsistent with experimental data.

## Competing interests

The authors declared that they have no competing interests.

## Authors’ contributions

MB and JR performed and designed research and wrote the paper. MD, AK, and EA performed research. JH designed research. WK wrote the paper. BO and BK designed research and wrote the paper. All authors read and approved the final manuscript.

## Supplementary Material

Additional file 1 This additional file contains all the supplementary figures along with their legends. Click here for file

## References

[B1] Goldbeter A Koshland DE Jr An amplified sensitivity arising from covalent modification in biological systems Proc Natl Acad Sci U S A 1981 78 11 6840 6844 10.1073/pnas.78.11.6840 6947258 PMC349147

[B2] Markevich NI Hoek JB Kholodenko BN Signaling switches and bistability arising from multisite phosphorylation in protein kinase cascades J Cell Biol 2004 164 3 353 359 10.1083/jcb.200308060 14744999 PMC2172246

[B3] Ferrell JE Jr Self-perpetuating states in signal transduction: positive feedback, double-negative feedback and bistability Curr Opin Cell Biol 2002 14 2 140 148 10.1016/S0955-0674(02)00314-9 11891111

[B4] Pomerening JR Sontag ED Ferrell JE Jr Building a cell cycle oscillator: hysteresis and bistability in the activation of Cdc2 Nat Cell Biol 2003 5 4 346 351 10.1038/ncb954 12629549

[B5] Sha W Moore J Chen K Lassaletta AD Yi CS Tyson JJ Sible JC Hysteresis drives cell-cycle transitions in Xenopus laevis egg extracts Proc Natl Acad Sci U S A 2003 100 3 975 980 10.1073/pnas.0235349100 12509509 PMC298711

[B6] Tyson JJ Csikasz-Nagy A Novak B The dynamics of cell cycle regulation Bioessays 2002 24 12 1095 1109 10.1002/bies.10191 12447975

[B7] Huang CY Ferrell JE Jr Ultrasensitivity in the mitogen-activated protein kinase cascade Proc Natl Acad Sci U S A 1996 93 19 10078 10083 10.1073/pnas.93.19.10078 8816754 PMC38339

[B8] Santos SD Verveer PJ Bastiaens PI Growth factor-induced MAPK network topology shapes Erk response determining PC-12 cell fate Nat Cell Biol 2007 9 3 324 330 10.1038/ncb1543 17310240

[B9] Mackeigan JP Murphy LO Dimitri CA Blenis J Graded mitogen-activated protein kinase activity precedes switch-like c-Fos induction in mammalian cells Mol Cell Biol 2005 25 11 4676 4682 10.1128/MCB.25.11.4676-4682.2005 15899869 PMC1140635

[B10] Harding A Tian T Westbury E Frische E Hancock JF Subcellular localization determines MAP kinase signal output Curr Biol 2005 15 9 869 873 10.1016/j.cub.2005.04.020 15886107

[B11] Inder K Harding A Plowman SJ Philips MR Parton RG Hancock JF Activation of the MAPK module from different spatial locations generates distinct system outputs Mol Biol Cell 2008 19 11 4776 4784 10.1091/mbc.E08-04-0407 18784252 PMC2575182

[B12] Ferrell JE Jr Machleder EM The biochemical basis of an all-or-none cell fate switch in Xenopus oocytes Science 1998 280 5365 895 898 10.1126/science.280.5365.895 9572732

[B13] McAdams HH Arkin A Stochastic mechanisms in gene expression Proc Natl Acad Sci U S A 1997 94 3 814 819 10.1073/pnas.94.3.814 9023339 PMC19596

[B14] Bar-Even A Paulsson J Maheshri N Carmi M O'Shea E Pilpel Y Barkai N Noise in protein expression scales with natural protein abundance Nat Genet 2006 38 6 636 643 10.1038/ng1807 16715097

[B15] Cohen-Saidon C Cohen AA Sigal A Liron Y Alon U Dynamics and variability of ERK2 response to EGF in individual living cells Mol Cell 2009 36 5 885 893 10.1016/j.molcel.2009.11.025 20005850

[B16] Niepel M Spencer SL Sorger PK Non-genetic cell-to-cell variability and the consEquationuences for pharmacology Curr Opin Chem Biol 2009 13 5–6 556 561 19833543 10.1016/j.cbpa.2009.09.015 PMC2975492

[B17] Spencer SL Gaudet S Albeck JG Burke JM Sorger PK Non-genetic origins of cell-to-cell variability in TRAIL-induced apoptosis Nature 2009 459 7245 428 432 10.1038/nature08012 19363473 PMC2858974

[B18] Chang HH Hemberg M Barahona M Ingber DE Huang S Transcriptome-wide noise controls lineage choice in mammalian progenitor cells Nature 2008 453 7194 544 547 10.1038/nature06965 18497826 PMC5546414

[B19] Raser JM O'Shea EK Noise in gene expression: origins, consEquationuences, and control Science 2005 309 5743 2010 2013 10.1126/science.1105891 16179466 PMC1360161

[B20] Wilkinson DJ Stochastic modelling for quantitative description of heterogeneous biological systems Nat Rev Genet 2009 10 2 122 133 10.1038/nrg2509 19139763

[B21] Pedraza JM Paulsson J Effects of molecular memory and bursting on fluctuations in gene expression Science 2008 319 5861 339 343 10.1126/science.1144331 18202292

[B22] Raj A Peskin CS Tranchina D Vargas DY Tyagi S Stochastic mRNA synthesis in mammalian cells PLoS Biol 2006 4 10 e309 10.1371/journal.pbio.0040309 17048983 PMC1563489

[B23] Yu J Xiao J Ren X Lao K Xie XS Probing gene expression in live cells, one protein molecule at a time Science 2006 311 5767 1600 1603 10.1126/science.1119623 16543458

[B24] Cai L Friedman N Xie XS Stochastic protein expression in individual cells at the single molecule level Nature 2006 440 7082 358 362 10.1038/nature04599 16541077

[B25] Colman-Lerner A Gordon A Serra E Chin T Resnekov O Endy D Pesce CG Brent R Regulated cell-to-cell variation in a cell-fate decision system Nature 2005 437 7059 699 706 10.1038/nature03998 16170311

[B26] Perez OD Nolan GP Phospho-proteomic immune analysis by flow cytometry: from mechanism to translational medicine at the single-cell level Immunol Rev 2006 210 208 228 10.1111/j.0105-2896.2006.00364.x 16623773

[B27] Taniguchi Y Choi PJ Li GW Chen H Babu M Hearn J Emili A Xie XS Quantifying E. coli proteome and transcriptome with single-molecule sensitivity in single cells Science 2010 329 5991 533 538 10.1126/science.1188308 20671182 PMC2922915

[B28] Shahrezaei V Swain PS Analytical distributions for stochastic gene expression Proc Natl Acad Sci U S A 2008 105 45 17256 17261 10.1073/pnas.0803850105 18988743 PMC2582303

[B29] Cohen AA Kalisky T Mayo A Geva-Zatorsky N Danon T Issaeva I Kopito RB Perzov N Milo R Sigal A Protein dynamics in individual human cells: experiment and theory PLoS One 2009 4 4 e4901 10.1371/journal.pone.0004901 19381343 PMC2668709

[B30] Paulsson J Berg OG Ehrenberg M Stochastic focusing: fluctuation-enhanced sensitivity of intracellular regulation Proc Natl Acad Sci U S A 2000 97 13 7148 7153 10.1073/pnas.110057697 10852944 PMC16514

[B31] Friedman N Cai L Xie XS Linking stochastic dynamics to population distribution: an analytical framework of gene expression Phys Rev Lett 2006 97 16 168302 17155441 10.1103/PhysRevLett.97.168302

[B32] Boykevisch S Zhao C Sondermann H Philippidou P Halegoua S Kuriyan J Bar-Sagi D Regulation of ras signaling dynamics by Sos-mediated positive feedback Curr Biol 2006 16 21 2173 2179 10.1016/j.cub.2006.09.033 17084704

[B33] Das J Ho M Zikherman J Govern C Yang M Weiss A Chakraborty AK Roose JP Digital signaling and hysteresis characterize ras activation in lymphoid cells Cell 2009 136 2 337 351 10.1016/j.cell.2008.11.051 19167334 PMC2662698

[B34] Prasad A Zikherman J Das J Roose JP Weiss A Chakraborty AK Origin of the sharp boundary that discriminates positive and negative selection of thymocytes Proc Natl Acad Sci U S A 2009 106 2 528 533 10.1073/pnas.0805981105 19098101 PMC2626737

[B35] Kholodenko BN Birtwistle MR Four-dimensional dynamics of MAPK information processing systems Wiley Interdiscip Rev Syst Biol Med 2009 1 1 28 44 10.1002/wsbm.16 20182652 PMC2826817

[B36] Markevich NI Tsyganov MA Hoek JB Kholodenko BN Long-range signaling by phosphoprotein waves arising from bistability in protein kinase cascades Mol Syst Biol 2006 2 61 17102806 10.1038/msb4100108 PMC1682027

[B37] Sauro HM Kholodenko BN Quantitative analysis of signaling networks Prog Biophys Mol Biol 2004 86 1 5 43 10.1016/j.pbiomolbio.2004.03.002 15261524

[B38] Birtwistle MR Kolch W Biology using engineering tools: the negative feedback amplifier Cell Cycle 2011 10 13 2069 2076 10.4161/cc.10.13.16245 21572255 PMC3154361

[B39] Sturm OE Orton R Grindlay J Birtwistle M Vyshemirsky V Gilbert D Calder M Pitt A Kholodenko B Kolch W The mammalian MAPK/ERK pathway exhibits properties of a negative feedback amplifier Sci Signal 2010 3 153 ra90 10.1126/scisignal.2001212 21177493

[B40] Fritsche-Guenther R Witzel F Sieber A Herr R Schmidt N Braun S Brummer T Sers C Bluthgen N Strong negative feedback from Erk to Raf confers robustness to MAPK signalling Mol Syst Biol 2011 7 489 21613978 10.1038/msb.2011.27 PMC3130559

[B41] Borisov N Aksamitiene E Kiyatkin A Legewie S Berkhout J Maiwald T Kaimachnikov NP Timmer J Hoek JB Kholodenko BN Systems-level interactions between insulin-EGF networks amplify mitogenic signaling Mol Syst Biol 2009 5 256 19357636 10.1038/msb.2009.19 PMC2683723

[B42] Kholodenko BN Negative feedback and ultrasensitivity can bring about oscillations in the mitogen-activated protein kinase cascades Eur J Biochem 2000 267 6 1583 1588 10.1046/j.1432-1327.2000.01197.x 10712587

[B43] French AR Tadaki DK Niyogi SK Lauffenburger DA Intracellular trafficking of epidermal growth factor family ligands is directly influenced by the pH sensitivity of the receptor/ligand interaction J Biol Chem 1995 270 9 4334 4340 10.1074/jbc.270.9.4334 7876195

[B44] Roepstorff K Grandal MV Henriksen L Knudsen SL Lerdrup M Grovdal L Willumsen BM van Deurs B Differential effects of EGFR ligands on endocytic sorting of the receptor Traffic 2009 10 8 1115 1127 10.1111/j.1600-0854.2009.00943.x 19531065 PMC2723868

[B45] Goentoro L Shoval O Kirschner MW Alon U The incoherent feedforward loop can provide fold-change detection in gene regulation Mol Cell 2009 36 5 894 899 10.1016/j.molcel.2009.11.018 20005851 PMC2896310

[B46] Nakakuki T Birtwistle MR Saeki Y Yumoto N Ide K Nagashima T Brusch L Ogunnaike BA Okada-Hatakeyama M Kholodenko BN Ligand-specific c-Fos expression emerges from the spatiotemporal control of ErbB network dynamics Cell 2010 141 5 884 896 10.1016/j.cell.2010.03.054 20493519 PMC2888034

[B47] Gius D Cao XM Rauscher FJ 3rd Cohen DR Curran T Sukhatme VP Transcriptional activation and repression by Fos are independent functions: the C terminus represses immediate-early gene expression via CArG elements Mol Cell Biol 1990 10 8 4243 4255 2115122 10.1128/mcb.10.8.4243-4255.1990 PMC360963

[B48] Kholodenko BN Cell-signalling dynamics in time and space Nat Rev Mol Cell Biol 2006 7 165 176 PMID: 16482094 10.1038/nrm1838 16482094 PMC1679905

[B49] Kiyatkin A Aksamitiene E Markevich NI Borisov NM Hoek JB Kholodenko BN Scaffolding protein Grb2-associated binder 1 sustains epidermal growth factor-induced mitogenic and survival signaling by multiple positive feedback loops J Biol Chem 2006 281 29 19925 19938 10.1074/jbc.M600482200 16687399 PMC2312093

[B50] Aksamitiene E Achanta S Kolch W Kholodenko BN Hoek JB Kiyatkin A Prolactin-stimulated activation of ERK1/2 mitogen-activated protein kinases is controlled by PI3-kinase/Rac/PAK signaling pathway in breast cancer cells Cell Signal 2011 23 11 1794 1805 10.1016/j.cellsig.2011.06.014 21726627 PMC3156300

[B51] Hartigan JA Hartigan PM The Dip Test of Unimodality Ann Stat 1985 13 1 70 84 10.1214/aos/1176346577

[B52] Hartigan PM Computation of the Dip Statistic to Test for Unimodality Appl Stat J R Stat Soc Ser C 1985 34 3 320 325

