# Supplementary material for: Emergence of bimodal cell population responses from the interplay between analog single-cell signaling and protein expression noise
Source: BMC Syst Biol. 2012 Aug 24;6:109. doi: 10.1186/1752-0509-6-109 (PMC3484110; doi:10.1186/1752-0509-6-109)
Supplement: Additional file 1 — This additional file contains all the supplementary figures along with their legends. [file 1752-0509-6-109-S1.ppt]

## Slide 1
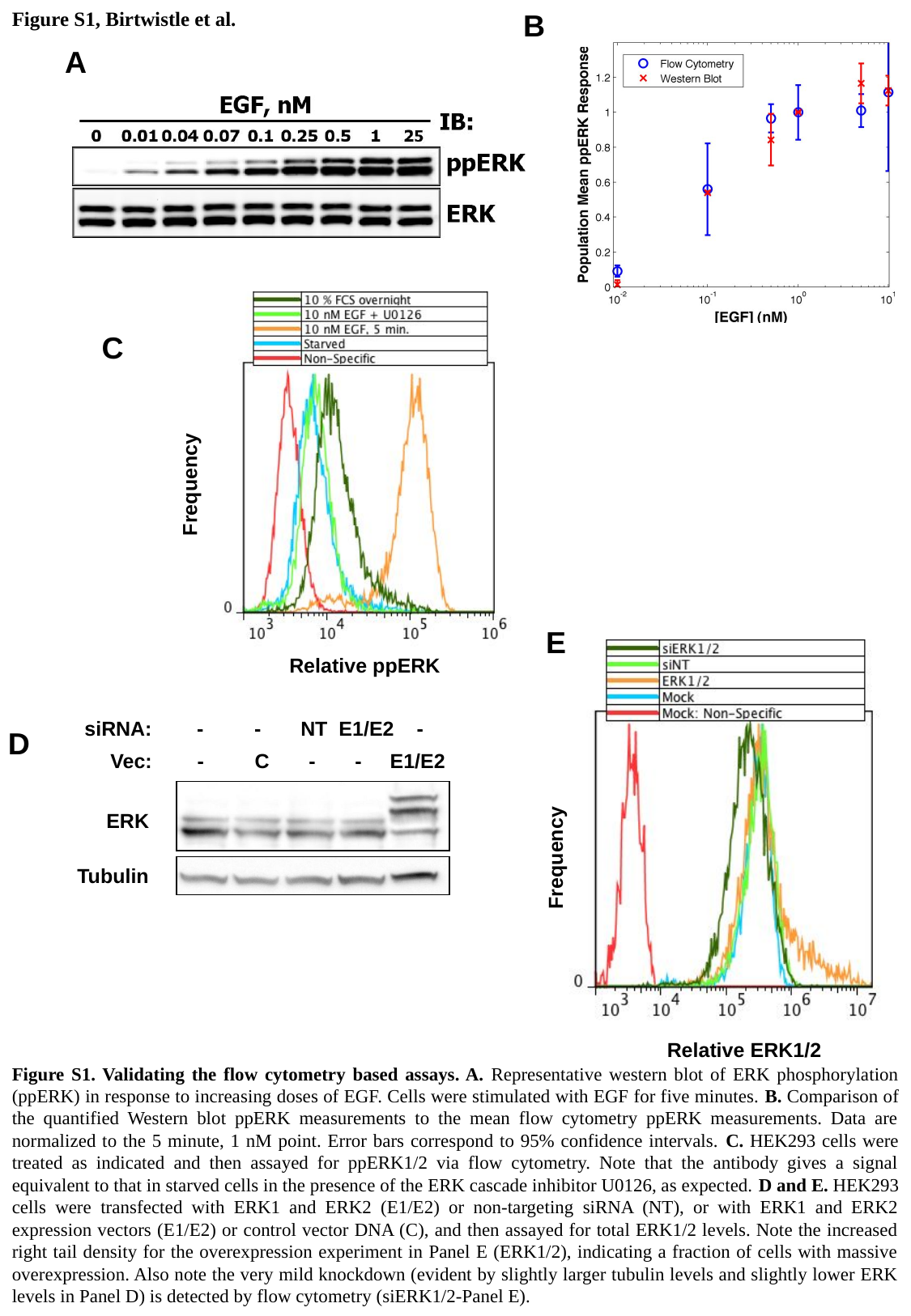

Figure S1, Birtwistle et al.
B
A
C
Frequency
E
 Relative ppERK
siRNA: - - NT E1/E2 -
D
Vec: - C - - E1/E2
 ERK
 Tubulin
Frequency
 Relative ERK1/2
Figure S1. Validating the flow cytometry based assays. A. Representative western blot of ERK phosphorylation (ppERK) in response to increasing doses of EGF. Cells were stimulated with EGF for five minutes. B. Comparison of the quantified Western blot ppERK measurements to the mean flow cytometry ppERK measurements. Data are normalized to the 5 minute, 1 nM point. Error bars correspond to 95% confidence intervals. C. HEK293 cells were treated as indicated and then assayed for ppERK1/2 via flow cytometry. Note that the antibody gives a signal equivalent to that in starved cells in the presence of the ERK cascade inhibitor U0126, as expected. D and E. HEK293 cells were transfected with ERK1 and ERK2 (E1/E2) or non-targeting siRNA (NT), or with ERK1 and ERK2 expression vectors (E1/E2) or control vector DNA (C), and then assayed for total ERK1/2 levels. Note the increased right tail density for the overexpression experiment in Panel E (ERK1/2), indicating a fraction of cells with massive overexpression. Also note the very mild knockdown (evident by slightly larger tubulin levels and slightly lower ERK levels in Panel D) is detected by flow cytometry (siERK1/2-Panel E).

## Slide 2
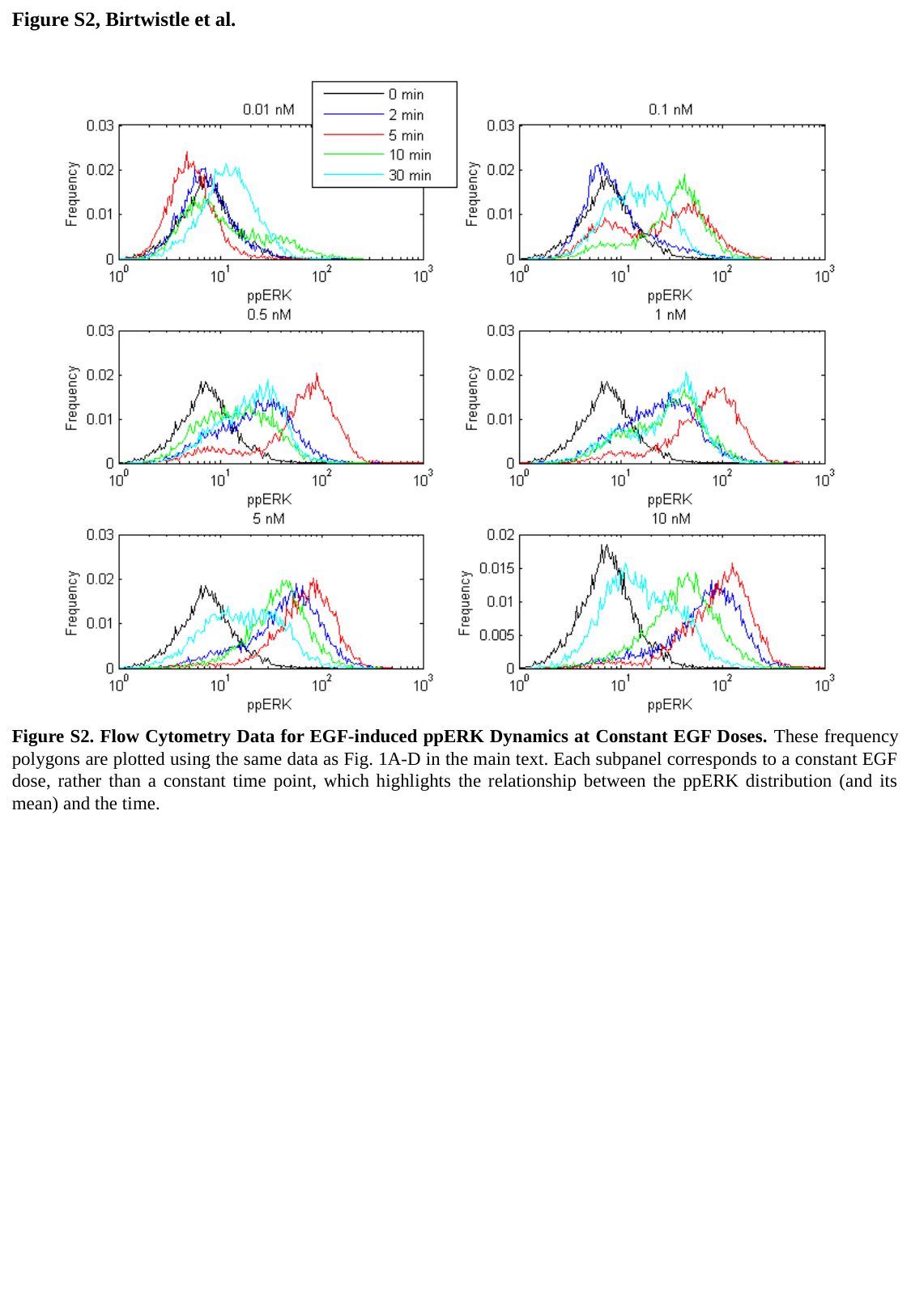

Figure S2, Birtwistle et al.
Figure S2. Flow Cytometry Data for EGF-induced ppERK Dynamics at Constant EGF Doses. These frequency polygons are plotted using the same data as Fig. 1A-D in the main text. Each subpanel corresponds to a constant EGF dose, rather than a constant time point, which highlights the relationship between the ppERK distribution (and its mean) and the time.

## Slide 3
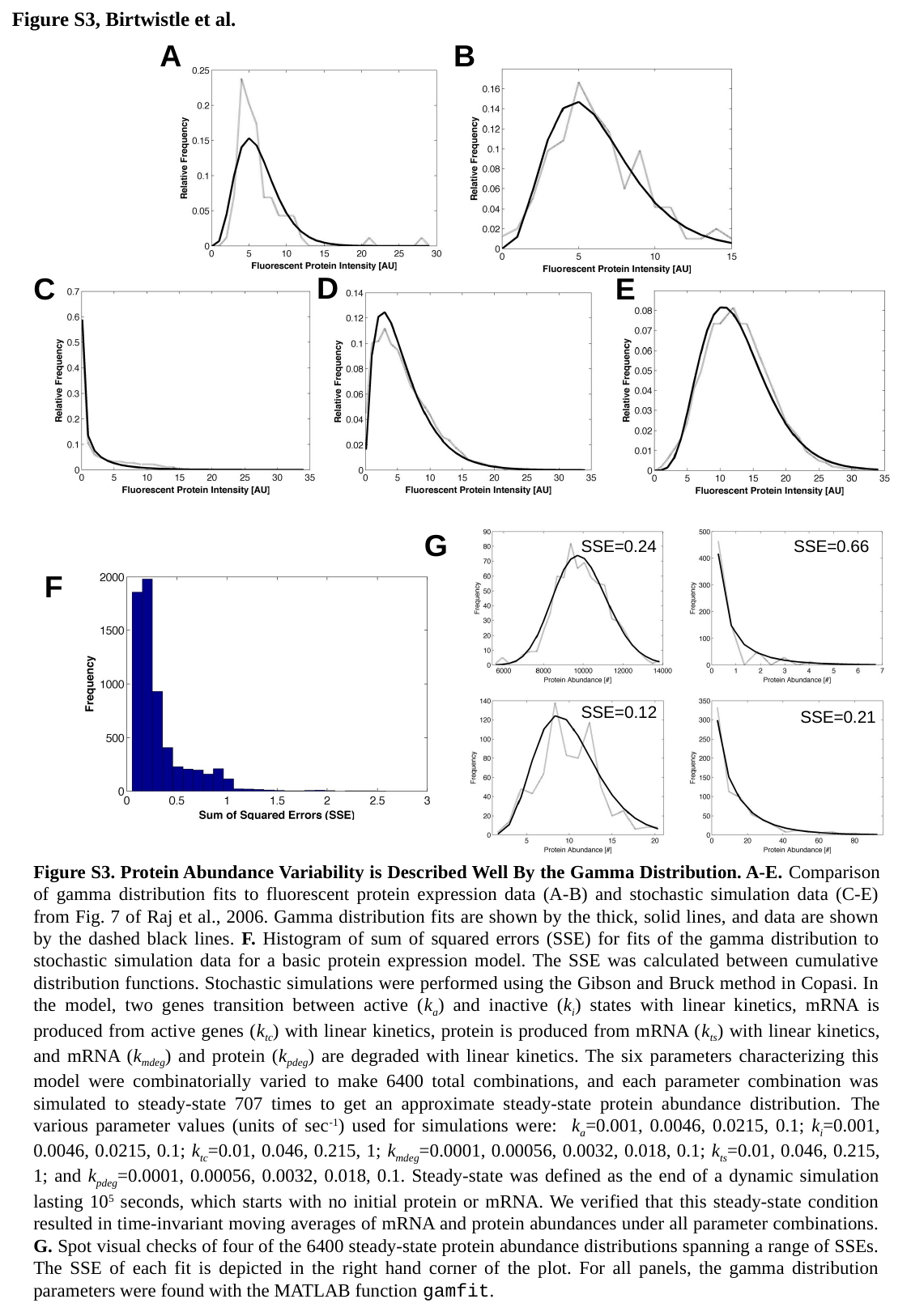

Figure S3, Birtwistle et al.
A
B
D
C
E
G
SSE=0.24
SSE=0.66
F
SSE=0.12
SSE=0.21
Figure S3. Protein Abundance Variability is Described Well By the Gamma Distribution. A-E. Comparison of gamma distribution fits to fluorescent protein expression data (A-B) and stochastic simulation data (C-E) from Fig. 7 of Raj et al., 2006. Gamma distribution fits are shown by the thick, solid lines, and data are shown by the dashed black lines. F. Histogram of sum of squared errors (SSE) for fits of the gamma distribution to stochastic simulation data for a basic protein expression model. The SSE was calculated between cumulative distribution functions. Stochastic simulations were performed using the Gibson and Bruck method in Copasi. In the model, two genes transition between active (ka) and inactive (ki) states with linear kinetics, mRNA is produced from active genes (ktc) with linear kinetics, protein is produced from mRNA (kts) with linear kinetics, and mRNA (kmdeg) and protein (kpdeg) are degraded with linear kinetics. The six parameters characterizing this model were combinatorially varied to make 6400 total combinations, and each parameter combination was simulated to steady-state 707 times to get an approximate steady-state protein abundance distribution. The various parameter values (units of sec-1) used for simulations were: ka=0.001, 0.0046, 0.0215, 0.1; ki=0.001, 0.0046, 0.0215, 0.1; ktc=0.01, 0.046, 0.215, 1; kmdeg=0.0001, 0.00056, 0.0032, 0.018, 0.1; kts=0.01, 0.046, 0.215, 1; and kpdeg=0.0001, 0.00056, 0.0032, 0.018, 0.1. Steady-state was defined as the end of a dynamic simulation lasting 105 seconds, which starts with no initial protein or mRNA. We verified that this steady-state condition resulted in time-invariant moving averages of mRNA and protein abundances under all parameter combinations. G. Spot visual checks of four of the 6400 steady-state protein abundance distributions spanning a range of SSEs. The SSE of each fit is depicted in the right hand corner of the plot. For all panels, the gamma distribution parameters were found with the MATLAB function gamfit.

## Slide 4
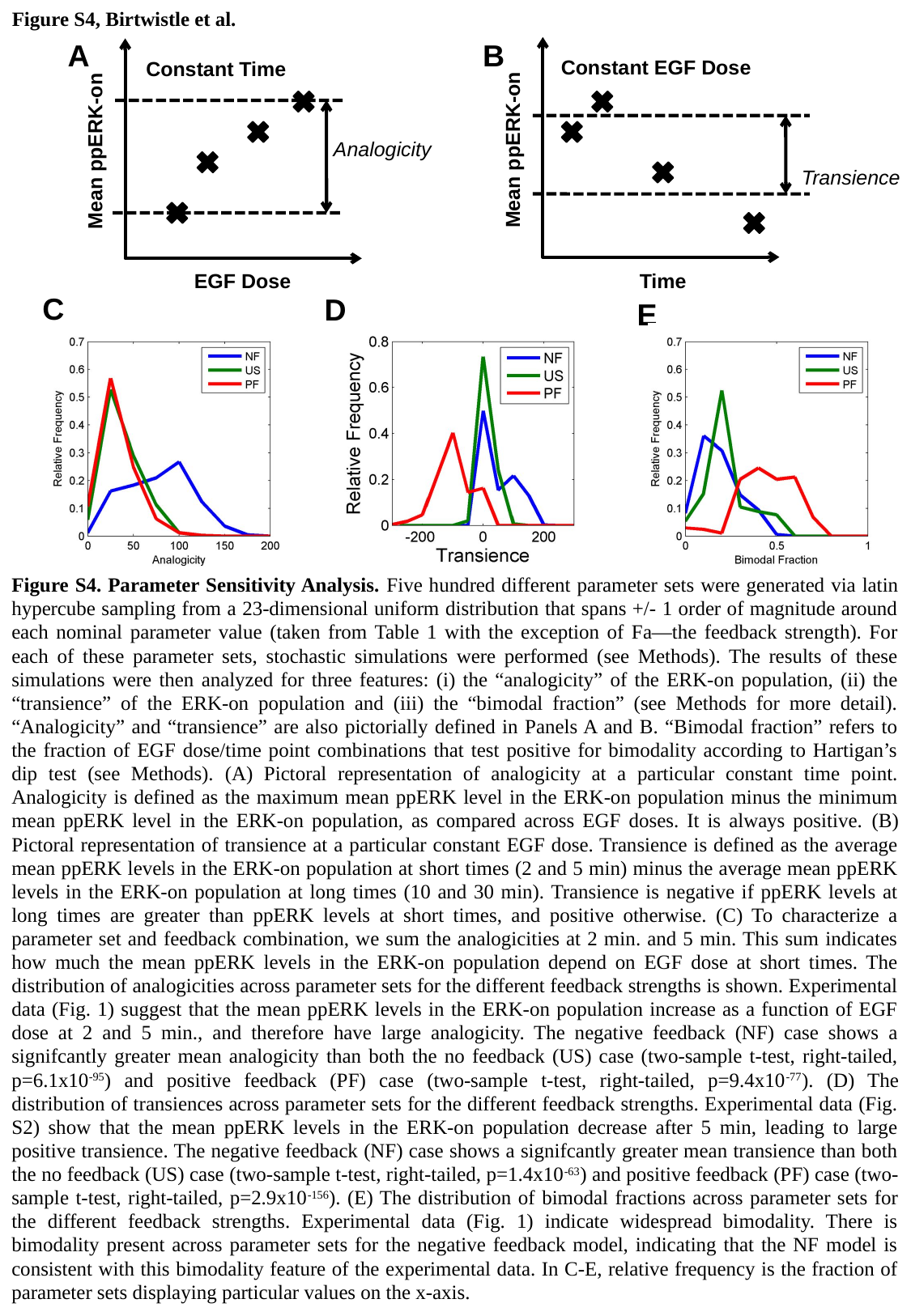

Figure S4, Birtwistle et al.
A
B
Constant EGF Dose
Constant Time
Mean ppERK-on
Mean ppERK-on
Analogicity
Transience
EGF Dose
Time
C
D
E
Figure S4. Parameter Sensitivity Analysis. Five hundred different parameter sets were generated via latin hypercube sampling from a 23-dimensional uniform distribution that spans +/- 1 order of magnitude around each nominal parameter value (taken from Table 1 with the exception of Fa—the feedback strength). For each of these parameter sets, stochastic simulations were performed (see Methods). The results of these simulations were then analyzed for three features: (i) the “analogicity” of the ERK-on population, (ii) the “transience” of the ERK-on population and (iii) the “bimodal fraction” (see Methods for more detail). “Analogicity” and “transience” are also pictorially defined in Panels A and B. “Bimodal fraction” refers to the fraction of EGF dose/time point combinations that test positive for bimodality according to Hartigan’s dip test (see Methods). (A) Pictoral representation of analogicity at a particular constant time point. Analogicity is defined as the maximum mean ppERK level in the ERK-on population minus the minimum mean ppERK level in the ERK-on population, as compared across EGF doses. It is always positive. (B) Pictoral representation of transience at a particular constant EGF dose. Transience is defined as the average mean ppERK levels in the ERK-on population at short times (2 and 5 min) minus the average mean ppERK levels in the ERK-on population at long times (10 and 30 min). Transience is negative if ppERK levels at long times are greater than ppERK levels at short times, and positive otherwise. (C) To characterize a parameter set and feedback combination, we sum the analogicities at 2 min. and 5 min. This sum indicates how much the mean ppERK levels in the ERK-on population depend on EGF dose at short times. The distribution of analogicities across parameter sets for the different feedback strengths is shown. Experimental data (Fig. 1) suggest that the mean ppERK levels in the ERK-on population increase as a function of EGF dose at 2 and 5 min., and therefore have large analogicity. The negative feedback (NF) case shows a signifcantly greater mean analogicity than both the no feedback (US) case (two-sample t-test, right-tailed, p=6.1x10^-95^) and positive feedback (PF) case (two-sample t-test, right-tailed, p=9.4x10^-77^). (D) The distribution of transiences across parameter sets for the different feedback strengths. Experimental data (Fig. S2) show that the mean ppERK levels in the ERK-on population decrease after 5 min, leading to large positive transience. The negative feedback (NF) case shows a signifcantly greater mean transience than both the no feedback (US) case (two-sample t-test, right-tailed, p=1.4x10^-63^) and positive feedback (PF) case (two-sample t-test, right-tailed, p=2.9x10-156). (E) The distribution of bimodal fractions across parameter sets for the different feedback strengths. Experimental data (Fig. 1) indicate widespread bimodality. There is bimodality present across parameter sets for the negative feedback model, indicating that the NF model is consistent with this bimodality feature of the experimental data. In C-E, relative frequency is the fraction of parameter sets displaying particular values on the x-axis.

## Slide 5
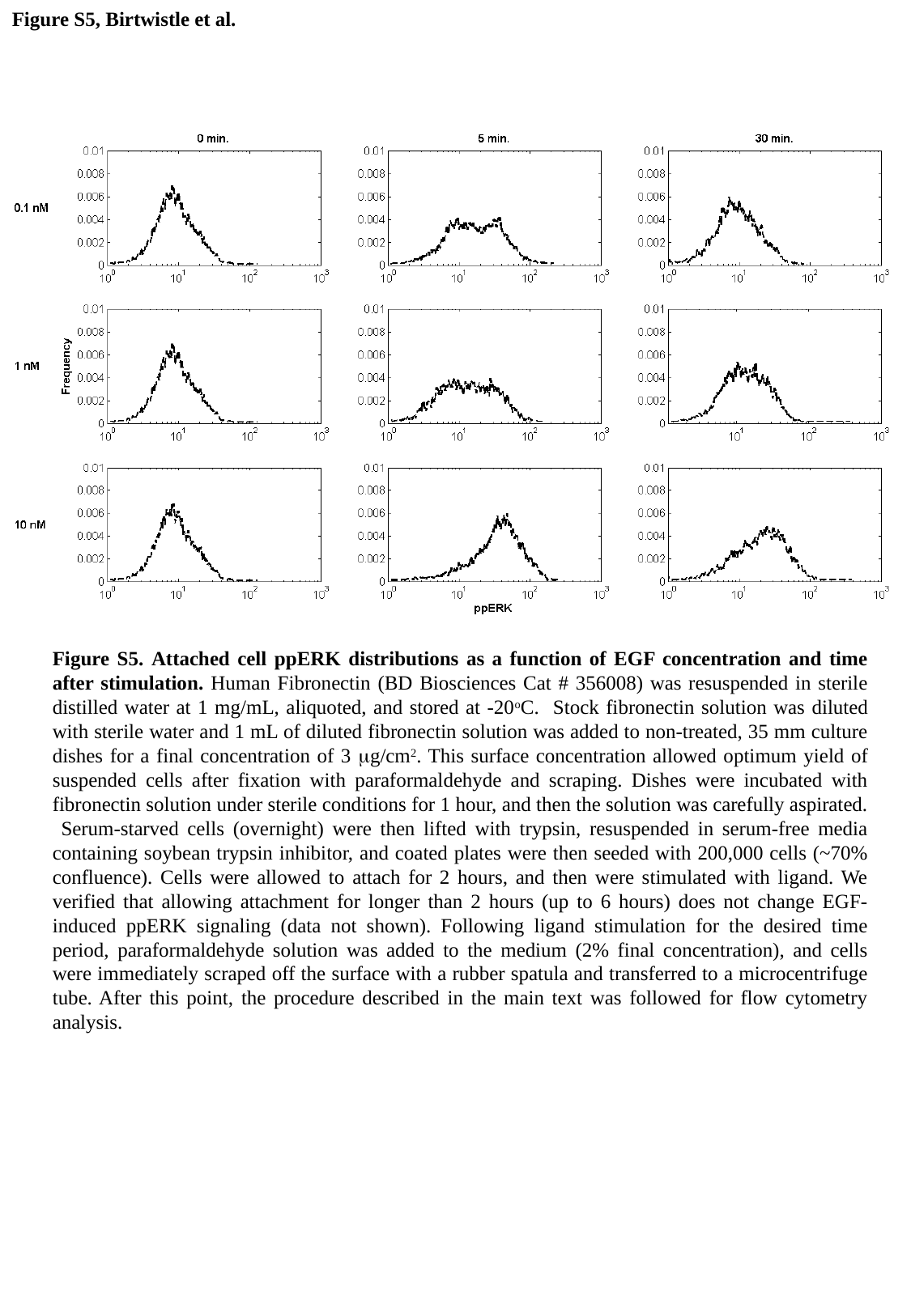

Figure S5, Birtwistle et al.
Figure S5. Attached cell ppERK distributions as a function of EGF concentration and time after stimulation. Human Fibronectin (BD Biosciences Cat # 356008) was resuspended in sterile distilled water at 1 mg/mL, aliquoted, and stored at -20oC. Stock fibronectin solution was diluted with sterile water and 1 mL of diluted fibronectin solution was added to non-treated, 35 mm culture dishes for a final concentration of 3 μg/cm2. This surface concentration allowed optimum yield of suspended cells after fixation with paraformaldehyde and scraping. Dishes were incubated with fibronectin solution under sterile conditions for 1 hour, and then the solution was carefully aspirated. Serum-starved cells (overnight) were then lifted with trypsin, resuspended in serum-free media containing soybean trypsin inhibitor, and coated plates were then seeded with 200,000 cells (~70% confluence). Cells were allowed to attach for 2 hours, and then were stimulated with ligand. We verified that allowing attachment for longer than 2 hours (up to 6 hours) does not change EGF-induced ppERK signaling (data not shown). Following ligand stimulation for the desired time period, paraformaldehyde solution was added to the medium (2% final concentration), and cells were immediately scraped off the surface with a rubber spatula and transferred to a microcentrifuge tube. After this point, the procedure described in the main text was followed for flow cytometry analysis.

## Slide 6
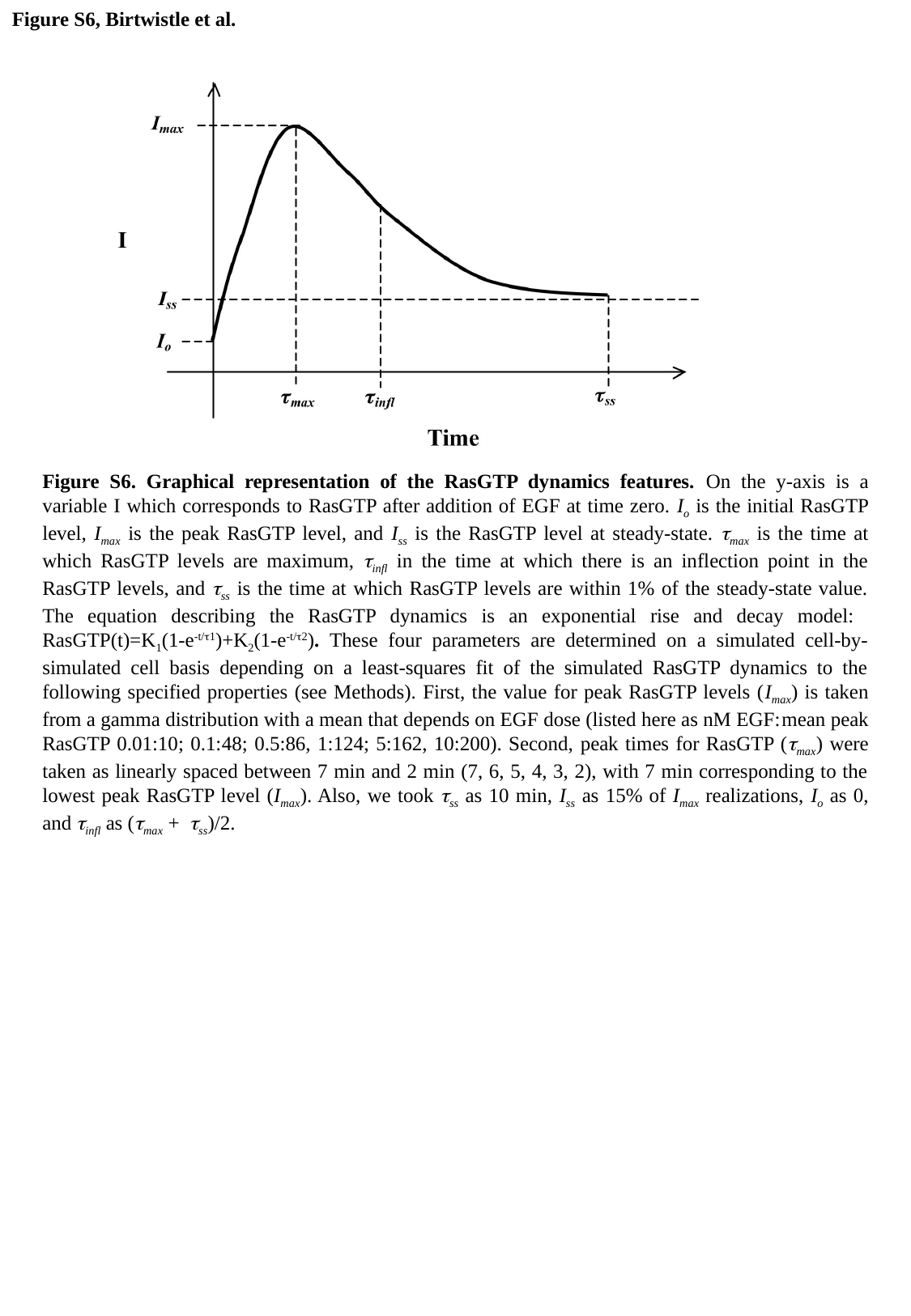

Figure S6, Birtwistle et al.
Figure S6. Graphical representation of the RasGTP dynamics features. On the y-axis is a variable I which corresponds to RasGTP after addition of EGF at time zero. Io is the initial RasGTP level, Imax is the peak RasGTP level, and Iss is the RasGTP level at steady-state. max is the time at which RasGTP levels are maximum, infl in the time at which there is an inflection point in the RasGTP levels, and ss is the time at which RasGTP levels are within 1% of the steady-state value. The equation describing the RasGTP dynamics is an exponential rise and decay model: RasGTP(t)=K1(1-e-t/τ1)+K2(1-e-t/τ2). These four parameters are determined on a simulated cell-by-simulated cell basis depending on a least-squares fit of the simulated RasGTP dynamics to the following specified properties (see Methods). First, the value for peak RasGTP levels (Imax) is taken from a gamma distribution with a mean that depends on EGF dose (listed here as nM EGF:mean peak RasGTP 0.01:10; 0.1:48; 0.5:86, 1:124; 5:162, 10:200). Second, peak times for RasGTP (τmax) were taken as linearly spaced between 7 min and 2 min (7, 6, 5, 4, 3, 2), with 7 min corresponding to the lowest peak RasGTP level (Imax). Also, we took ss as 10 min, Iss as 15% of Imax realizations, Io as 0, and infl as (τmax + ss)/2.
